# Evaluation of a nurse practitioner-physician task-sharing model for multidrug-resistant tuberculosis in South Africa

**DOI:** 10.1371/journal.pone.0182780

**Published:** 2017-08-04

**Authors:** Jason E. Farley, Norbert Ndjeka, Ana M. Kelly, Erin Whitehouse, Simmi Lachman, Chakra Budhathoki, Kelly Lowensen, Ellie Bergren, Hloniphile Mabuza, Ntombasekhaya Mlandu, Martie van der Walt

**Affiliations:** 1 School of Nursing, Johns Hopkins University, Baltimore, Maryland, United States of America; 2 Republic of South Africa Department of Health, Pretoria, South Africa; 3 School of Nursing, Columbia University, New York, New York, United States of America; 4 Murchison District Hospital, Port Shepstone, South Africa; 5 Center for Disease Control and Prevention, Atlanta, Georgia, United States of America; 6 Johns Hopkins Health Education-South Africa (JHHESA), Pretoria, South Africa; 7 Medical Research Council, Pretoria, South Africa; Waseda University, JAPAN

## Abstract

**Background:**

Treatment success rates for multidrug-resistant tuberculosis (MDR-TB) in South Africa remain close to 50%. Lack of access to timely, decentralized care is a contributing factor. We evaluated MDR-TB treatment outcomes from a clinical cohort with task-sharing between a clinical nurse practitioner (CNP) and a medical officer (MO).

**Methods:**

We completed a retrospective evaluation of outcomes from a prospective, programmatically-based MDR-TB cohort who were enrolled and received care between 2012 and 2015 at a peri-urban hospital in KwaZulu-Natal, South Africa. Treatment was provided by either by a CNP or MO.

**Findings:**

The cohort included 197 participants with a median age of 33 years, 51% female, and 74% co-infected with HIV. The CNP initiated 123 participants on treatment. Overall MDR-TB treatment success rate in this cohort was 57.9%, significantly higher than the South African national average of 45% in 2012 (p<0·0001) and similar to the provincal average of 60% (p = NS). There were no significant differences by provider type: treatment success was 61% for patients initiated by the CNP and 52.7% for those initiated by the MO.

**Interpretation:**

Clinics that adopted a task sharing approach for MDR-TB demonstrated greater treatment success rates than the national average. Task-sharing between the CNP and MO did not adversely impact treatment outcome with similar success rates noted. Task-sharing is a feasible option for South Africa to support decentralization without compromising patient outcomes. Models that allow sharing of responsibility for MDR-TB may optimize the use of human resources and improve access to care.

## Introduction

Multidrug-resistant tuberculosis (MDR-TB) is a global epidemic, threatening progress towards the World Health Organization’s (WHO) Global Plan to Stop TB [1]. According to the most recent WHO report, MDR-TB, defined as TB with resistance to isoniazid and rifampin, occurred in over 480,000 people in 2015 [2]. Of these, less than one quarter are known to be receiving treatment [2]. Global treatment success rates for MDR-TB are exceptionally poor with estimates ranging between 49 and 68% compared to 83% for drug-susceptible TB [2]. In South Africa, MDR-TB treatment success is consistent with global norms with 50% of patients having a successful outcome [2]. HIV co-infection complicates MDR-TB treatment and worsens outcomes in South Africa where co-infection may exceed 70% [2–3]. Weaknesses in the healthcare system including lengthy treatment delays are a large part of low success rates [3–4].

Many countries have opted for centralized, hospital-based care in regional referral centers to manage drug-resistant TB. This strategy falls short in high-prevalence areas where many participants are concentrated in rural areas with limited hospital bed capacity [4]. In 2011, The South African National Department of Health (NDoH) recommended decentralization of MDR-TB treatment service to outpatient settings [5]. Decentralization required hospitals to down-refer after treatement initiation to lower level care settings to facilitate care closer to home. This model has shown improvements in patients’ overall care outcomes using physicians to provide care at decentralized centers [4,6]. Unfortunately, scarce resources, treatment related adverse effects and medical officer shortages pose challenges to implementing this model to scale [7–8]. Further, this policy change was enacted with limited evidence to support true decentralization to non-hospital settings or with non-physician providers.

One potential solution to this human resource challenge is task-sharing, a hybrid and more cooperative form of task shifting, which is defined as the movement of medical officer-driven clinical tasks to nurses with appropriate training [9]. Task-shifting has been implemented successfully to expand access to care for people living with HIV across sub-Saharan Africa [10–17]. Previous research on task-shifting concluded that it was an effective strategy for addressing human resource shortages for HIV treatment and care [18–21]. The most common intervention was the delegation of initiating and monitoring antiretroviral therapy (ART) from physicians to nurses. Nurse-led models of care have demonstrated equal or, in some cases, improved outcomes for HIV-infected participants compared to standard, physician-led models of care. Equivalent or improved outcomes in the nurse-led models included increased access to treatment [14,17,18], retention [10,11,14,16,20–21], patient acceptability [17,20–21], improved viral load [10,12,15,19,21], decreased mortality [10–21], and reduced costs [13,18]. Similar benefits from utilizing nurse prescribers to alleviate human resource challenges have also been demonstrated in other patient populations [22]. Task-sharing is a model similar to task-shifting, but follows a shared approach to clinical care where healthcare workers share responsibility on some activities while maintaining pre-existing tasks to share the workload [14,23–24]. This study evaluated MDR-TB treatment outcomes from a task-sharing intervention between a medical officer (MO) and clinical nurse practitioner (CNP) as a potential human resource solution to improve access to care and found that task-sharing is an effective model for delivering safe patient care.

## Materials and methods

### Study site

A single, peri-urban district hospital with a stand alone MDR-TB treatment unit in KwaZulu-Natal was chosen. The MDR-TB complex, included a 40 bed MDR-TB inpatient unit equally divided between male and female wards. The outpatient clinic for MDR-TB is situated adjacent to the inpatient ward and the hospital’s outpatient HIV clinic. The majority of patients at the site are admitted to the hospital ward for treatment initiation, however this varied based on bed capacity. The site follows the South African national treatment guidelines for clinical management. The catchment population for the hospital is noted to be 226,008 with six feeder primary care clinics. It was the only MDR-TB treatment initiation site in the district at the time of the study [25].

### Clinical standard of care

Diagnosis of drug-resistant tuberculosis was made using standard culture-based approaches with *Mycobacterium* growth indicator tubes (MGIT) followed by drug sensitivity testing (DST) on positive samples. All samples were processed through the National Health Laboratory System. Patients diagnosed with MDR-TB received the clinical standard of care for MDR-TB treatment in KwaZulu-Natal, South Africa, which for this area of the country includes a six-drug regimen [6]. Treatment includes a weight-based dosing of an injectable aminoglycoside (i.e., kanamycin) along with five oral medications: pyrazinamide, ethambutol, moxifloxacin, terizidone, and ethionamide. The six-drug regimen continues for four months after the point of sputum culture conversion with a minimum treatment duration of six months (i.e., intensive phase). When the injectable regimen is completed, the continuation phase then follows with the same oral regimen for a total treatment course of 24 months. Pyridoxine is a standard part of the regimen for the majority of patients, with dosages ranging between 25mg and 150mg for the prevention of peripheral neuropathy.

### Study enrollment

Participants were included in the programmatically-based observational cohort if they started MDR-TB treatment between January 3, 2012 and December 31, 2012. Upon cohort completion, all participant charts were reviewed until the point of outcome, which included all treatment completion and cure patients up to 24 months of follow-up. The final completion of treatment on all participants was January 2015. In May 2015, the treatment outcomes for all participants, received a second review and verification by the study team. The study was approved by The Johns Hopkins University Institutional Review Board in Baltimore, Maryland, USA and the Medical Research Council Ethics Committee in Pretoria, South Africa. The study was reviewed and approved by the Centers for Disease Control and Prevention under the protocol entitled, “CO-INFECT Project: Nurse Case Management”.

### Clinical procedures and assignment to MO or CNP

All patients presenting for MDR-TB care with culture-based evidence of isoniazid and rifampicin resistance were eligible to receive care from either provider and assignment was based on availability of the next provider. Study investigators had no role in treatment assignment. Participants, however, who presented with a baseline body mass index (BMI) less than 18 kg/m^2^ automatically received care from the medical officer due to severity of illness on presentation, which was a programmatically-based decision. All participants were given the option to be followed by the MO during the standard hospital consent process for treatment if they did not wish to have their care completed by the CNP.

Clinical duties for both providers included all required care during the treatment period, including inpatient services. Face to face and telephonic clinical consultation with the medical officer was available and completed as needed from the CNP throughout the course of treatment. In general, however, each clinician managed their assigned caseload unless out of the office, at which time the other clinician provided back-up clinical coverage for the absent member’s caseload. As part of current standardized care in South Africa, all patients with MDR-TB sign a one-page indemnity form for treatment that details the treatment regimen, provider options and basic expectations associated with the treatment course.

Treatment outcomes based on the South African NDoH guidelines are determined by sputum culture results and include treatment success (cure/completion of therapy), death (for any cause), failure (worsening drug resistance necessitating change in treatment), and default, which is now known as loss to follow-up (interruption of treatment for two consecutive months) [6]. Process indicators include the time to event for the outcome and length of inpatient stay. For the purposes of this study, clinical treatment outcome as defined by the treating provider was used as the categorical study outcome. Demographic, clinical and process variables were collected from the study participants.

### Pre-intervention clinical preparation of CNP and MO

The CNP’s prerequisite training included an advanced diploma in primary healthcare management (i.e., 18-month course of study after basic registered nurse training) and training in nurse-initiation and management of anti-retroviral therapy (NIMART). The MO’s prerequisite training included a 5-year Bachelor’s of Medicine (MBBCh) with an additional year of community service and training in HIV clinical management through an Advanced Post Graduate Diploma. The MO had also completed a one-week skills building short-course developed by The Johns Hopkins University School of Nursing in the diagnosis, treatment, and management of MDR-TB. Both the MO and CNP had extensive prior experience in the study hospital site. After hire, the CNP participated in the same one-week short course as the MO followed by an intensive one-week physical exam and health assessment skills update in the MDR-TB unit. Study team members with substantial MDR-TB clinical knowledge use a standardized competency-based tool, developed by the study team, to validate the CNPs baseline clinical skills through direct observation. After this evaluation the CNP received a one-month period of clinical mentoring by MOs at two regional drug-resistant TB centers in the province. Ancillary training and clinical mentoring were provided to the CNP during this period of supervised clinical mentorship in the following MDR-TB activities: chest x-ray evaluation and interpretation, laboratory monitoring, adverse treatment effects and their management as well as audiometry monitoring and interpretation.

### Statistical analysis

We computed descriptive statistics of the sample overall, and by provider type (CNP vs. MO). Medians (interquartile range, IQR) were calculated for skewed continuous variables and frequencies (%) were calculated for categorical variables. A Wilcoxon rank-sum test was used to compare the two groups (CNP vs. MO) for continuous patient characteristics and outcome variables and a Chi-square test was used to compare proportion of treatment outcome type between provider types. A one-sample z-test was used to compare the success rate in this cohort with the national programmatic outcomes data from South Africa. The treatment outcome was analyzed as proportion of outcome and also as time to an event. A multivariate Cox proportional hazards model was used to examine the association between provider type and time to a treatment outcome to evaluate type-specific hazards controlling for age, sex and baseline HIV status [26]. Significance was evaluated at alpha = 0.05 for all two-sided statistical tests.

## Results

Two-hundred twelve participants presented with MDR-TB for treatment at this site. Fifteen participants (7%) transferred their care to another facility making them ineligible for the final cohort analysis. One hundred ninety-seven participants, 101 (51.3%) female, with a median age of 33 years (IQR = 26–40) and a median body mass index (BMI) of 19.7 (IQR = 16.4–22.7) were enrolled. HIV status at enrollment was known for all but one patient with 145 (74%) co-infected with HIV (median CD4 count 250 cells per μL, IQR = 141–385) and of those, 108 (74.5%) were on ART at enrollment. The majority of participants (163, 82.7%) were admitted to the hospital for treatment initiation. BMI was a significantly different (p = 0.049) variable at baseline between the two providers (Table 1) which matched enrollment criteria. Of the 197 participants, 123 (62.4%) were initiated and followed on treatment by the CNP and 74 (37.6%) initiated by the MO with a similar time to treatment initiation (Table 1). Overall, patient characteristcs were nicely balanced, except as planned for BMI, between the two groups (Table 1). No participants requested reassignment during the period of follow-up.

**Table 1:** Patient characteristics by provider type.

Characteristics	Overall (n = 197)	CNP (n = 123)	MO (n = 74)	
	N, Median (IQR) or n (%)^†^	N, Median (IQR) or n (%)^†^	N, Median (IQR) or n (%)^†^	P-value*
Age (years)	197, 33.0	123, 34.0	74, 31.5	0.226
	(26–40)	(26–41)	(26–38)	
Female	101	61	40	0.544
	(51.3%)	(49.6%)	(54.1%)	
BMI	140, 19.7	116, 19.9	24, 16.9	0.049
	(16.4–22.7)	(16.9–22.8)	(14.4–21.6)	
Weight (kg)	193, 53.5	123, 54	70, 52	0.292
	(46.2–60)	(47–60)	(45–60)	
Unemployed^‡^	138/185	86/117	52/68	0.655
	(74.6%)	(73.5%)	(76.5%)	
HIV positive at baseline	145/196	90/123	55/73	0.738
	(74.0%)	(73.2%)	(75.3%)	
ART use at baseline among HIV positives	108/145	65/90	43/55	0.424
	(74.5%)	(72.2%)	(78.2%)	
CD4 count at baseline	137, 250	86, 225.5	51, 284	0.795
	(141–385)	(124–412)	(157–378)	
Time from MDRTB diagnosis to treatment initiation (days)	197, 71	123, 72	74, 71	0.685
	(51–92)	(50–100)	(51–88)	
Community/Admission				
Inpatient Unit	163 (82.7%)	101 (82.1%)	62 (83.8%)	0.406
Community	12 (6.1%)	6 (4.9%)	6 (8.1%)	
Admitted Later	22 (11.2%)	16 (13.0%)	6 (8.1%)	

*P-value from Wilcoxon rank-sum test for continuous variables, from Chi-square test for categorical variables; Unit = patients who initiated treatment as an inpatient; community = patients who initiated treatment as an outpatient; admitted later = patients who initiated treatment as an outpatient due to lack of bed availability, but who were subsequently admitted when a bed became available.

^†^Median (IQR = Q1-Q3) for continuous variables; n (%) for categorical variable, denominator (N) provided if different from given in header.

^‡^6 students excluded (from employment categories)

### MDR-TB treatment outcomes

**Fig 1:**
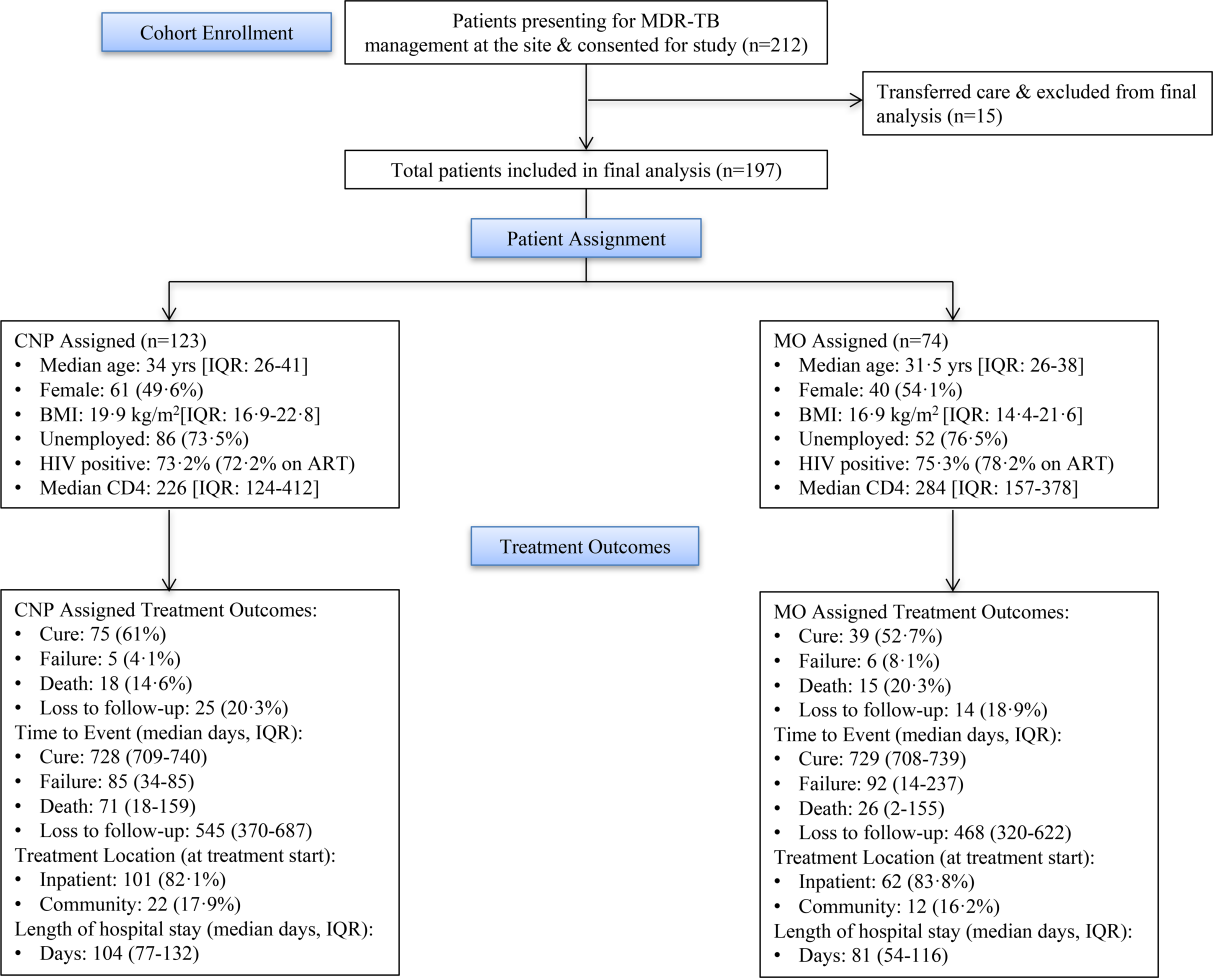
Participant enrollment and MDR-TB treatment outcomes between CNP and MO.

Treatment success for the cohort was 57.9% and significantly higher than the 45% national norm (z = 3.70, p<0.0001) for the 2012 South African cohort amongst 8,435 patients and similar to the 60% success rate for KwaZulu-Natal province amongst 3,190 patients (p = NS). Eleven (5.6%) participants experienced treatment failure, 33 (16.8%) died, and 39 (19.8%) were lost to follow-up (Fig 1). Comparing treatment outcomes based on provider type, success was documented for 75 (61%) of the CNP-assigned participants and 39 (52.7%) of the MO-assigned participants, with similar success rates. Median length of stay (days) was shorter in the MO group (81, IQR = 54–116) than CNP group (104, IQR = 77–132, p = 0.015) (Table 2). In the multivariate model, hazard of a treatment outcome as a competing event was not significantly different by provider type controlling for age, sex and HIV status (Table 3). There was no significant difference by HIV status for either cure, failure or lost to follow-up; however, hazard of death was 2.75 times higher in HIV positive patients than negative patients after controlling for age, sex and provider type (p = 0.044). Age was also associated with an increased hazard (hazard ratio [HR] 1.06, p = 0.001) for death, with 6% increase in hazard of death with an additional year of patient age after adjusting for sex, HIV status and provider type.

**Table 2:** Patient outcome data by provider type.

Characteristics	Overall (n = 197)	CNP (n = 123)	MO (n = 74)	
	N, Median (IQR) or n (%)^†^	N, Median (IQR) or n (%)^†^	N, Median (IQR) or n (%)^†^	P-value*
Time from Tx start to culture conversion (days)	160, 56	106, 56	54, 58	0.840
	(29–92)	(29–92)	(28–92)	
Length of intensive phase (days)	153, 196	100, 196	53, 198	0.632
	(181–212)	(186–211)	(180–215)	
Time from Tx start to Outcome (days)				
Success	728 (709–739)	728 (709–740)	729 (708–739)	0.363 (HR: 0.83‡)
Failure	85 (18–188)	85 (34–85)	92 (14–237)	0.200 (HR: 2.17‡)
Death	46 (16–155)	71 (18–159)	26 (2–155)	0.243 (HR: 1.50‡)
Loss to follow-up	524 (331–661)	545 (370–687)	468 (320–622)	0.903 (HR: 1.04‡)
Clinical Outcome				0.420
Success	114 (57.9%)	75 (61.0%)	39 (52.7%)	
Failure	11 (5.6%)	5 (4.1%)	6 (8.1%)	
Death	33 (16.8%)	18 (14.6%)	15 (20.3%)	
Loss to follow-up	39 (19.8%)	25 (20.3%)	14 (18.9%)	
Length of stay (inpatient days)	176, 95	112, 104	64, 81	0.015
	(61–122)	(77–132)	(54–116)	

*P-value from Wilcoxon rank-sum test for continuous variables, and from Chi-square test for categorical variables; Tx = treatment; Success = cure and treatment completion

^†^Median (IQR = Q1-Q3) for continuous variables; n (%) for categorical variable

‡ HR: Hazard ratio from unadjusted MO to CNP comparison from a time to event model

**Table 3:** Multivariate Cox proportional harzard model on time to one of the four treatment outcomes for comparison of cause-specific hazard between provider type.

	Success			Failure			Default			Death		
	HR	95% CI	P-value*	HR	95% CI	P-value*	HR	95% CI	P-value*	HR	95% CI	P-value*
Age (years)	1.00	0.99, 1.02	0.641	0.98	0.92, 1.04	0.491	0.97	0.94, 1.01	0.143	1.06	1.02, 1.09	0.001
Sex Female Male (ref)	1.02	0.70, 1.50	0.902	0.36	0.09, 1.47	0.155	0.91	0.46, 1.79	0.775	0.57	0.28, 1.16	0.120
HIV status Positive Negative (ref)	0.85	0.55, 1.31	0.457	0.40	0.11, 1.41	0.153	1.14	0.53, 2.44	0.733	2.75	1.03, 7.36	0.044
Provider^†^ MO CNP (ref)	0.84	0.55, 1.28	0.413	2.43	0.73, 8.05	0.147	1.08	0.56, 2.09	0.819	1.70	0.85, 3.41	0.134

*P-value for Wald test

^†^ Adjusting for age, sex, and baseline HIV status

MO, medical officer

CNP, clinical nurse practitioner

## Discussion

In a prospective, programmatically-based, observational cohort, in a peri-urban site with over 70% HIV co-infection, a task-sharing approach for MDR-TB demonstrated greater treatment success rates (57.9%) than the national average (45%).Task-sharing between the providers did not adversely impact treatment outcome. Process indicators and clinical outcomes were similar between providers, with the one exception of length of stay, which was shorter in the MO-led group. These findings demonstrate that nurse-led treatment did not compromise patient care. These findings support the potential for task-sharing for MDR-TB treatment initiation and management between medical officers and clinical nurse practitioners to work in some settings. Participants with HIV and increasing age had an increased hazard for death, which was independent of provider assignment and consistent with previously published literature [2–4].

These findings add further support to previous studies conducted in under-resourced African settings that utilize nurse-led models of care to manage HIV infection [10–17]. Decentralization of drug-resistant TB care, as a key policy platform of The South African National Department of Health, is designed to improve timely access to treatment. In this model, the use of culture-based diagnostic methods was the main reason for a median time to treatment initiation of 71 days. Presently, the country has fully implemented GeneXpert testing with results available in less than 3 days for most sites. As such, we urgently need data to inform current time between diagnosis and treatment initiation to support the importance of the expansion of such task-sharing programs. Decentralized, MO-led models of care are demonstrating improved outcomes in South Africa when compared to MO-led centralized approaches [27]; however, the necessary human resources to ensure all treatment sites have a MO do not exist in much of the country. A task-sharing nurse based model may alleviate this gap in human resources while maintaining high quality care.

While this evaluation of a CNP-MO MDR-TB program is the first of its kind and demonstrates programmatic outcomes exceeding those at the country level, limitations exist. The study was limited to retrospective abstraction of clinical records and documentation describing the clinical care for each patient. While this may have limited impact on the final treatment outcome determination, it limits the standardization of the type, frequency and preciseness one might anticipate in a more controlled research evaluation. To avoid missing data–mainly from lost or damaged medical files—our research team visited the site on a quarterly basis to retrospectively abstract data from the medical record. We also know that the treating clinical team was limited, and consisted of only one MO and one CNP initiating and managing MDR-TB treatment at the clinic, a circumstance not unusual in the South African context. This resulted, however, in the MO and CNP supporting one another during periods of illness or extended leave of absence. Although this limited the granular nature of our comparison, we felt that this improved generalizability and was ultimately a strength of the task-sharing model in a real-world setting, as the sharing of patient responsibility benefited both clinicians. The cross coverage needs that arose from the real world human resource challenge that exist resulted in approximately four months where the CNP was the sole clinician and another two months where the MO was the sole clinician. In both circumstances, participants received care outside of their original patient assignment. It is important to note that without such a model the MDR-TB program would have been without a trained MDR-TB care provider for approximately four months in an MO-only model. In the year prior to the programmatic implementation of this approach, this site experienced a period of more than six months without a trained clinician responsible for managing MDR-TB participants. It is important to note that no increase in death, default nor failure was noted during the time the CNP provided sole coverage. While the CNP can take on much of the patient burden thereby freeing the MO for care of more complicated MDR-TB cases, this circumstance channels sicker patients into the MO’s care and may bias outcome comparisons. In this cohort, the MO did manage participants who were significantly underweight (BMI < 18) on presentation to care, but this did not lead to differences in treatment outcome. It does likely account for the slightly lower success rates and lower inpatient days for the MO. Another possible explanation for this finding is that the MO had greater clinical experience and therefore felt more comfortable releasing the patient from the hospital earlier. Finally, as an observational study, this descriptive analysis does not offer the necessary evidence to support large scale implementation; however these data are promising and suggest the need for additional randomized evaluations.

Task-sharing is a feasible option for the peri-urban areas in South Africa to support decentralization efforts without compromising patient outcomes. Further evaluation through randomized controlled trials is required to more fully determine the impact of nurse-led models of MDR-TB care. As access to care remains a priority, the South African NDoH is presently decentralizing care to community health centers using the nurse-led model of care that has been presented in this study [28–29].
